# Literary Notes

**Published:** 1910-01-15

**Authors:** 


### Literary Notes.

Mr. Cavendish Morton, who has had many years' experience of the stage in the companies of Mr. Tree and Mr. Forbes Robertson, has written a book for Messrs. Black on the *Art of Theatrical Make-up*. The volume is illustrated by striking photographs.

"Natural and Social Morals," by Professor Carveth Read, M.A., is an attempt to explain the principles of morality in as close a relation as possible to human life. It takes account of the historical systems of ethics, of recent researches into the development of the human race, and of our present circumstances. The volume is published by Messrs. A. and C. Black.

Messrs. J. and A. Churchill, of 7 Great Marlborough Street, London, W., have issued their latest catalogue. They have been successful in their endeavour to arrange it so that it can be most easily consulted for reference. The books have been classified under 34 heads, and many specimen illustrations are given. The index to authors and list of volumes are long and varied, and indicate a wide range of literary work in medicine and science. The catalogue is attractive and well arranged, and shows the continued enterprise of this important firm of medical publishers.

The Sleeping Sickness Bureau has published an official Bibliography of sleeping sickness. This work has been compiled by Major C. A. Thimm, and contains an almost complete list of references to the published works on sleeping sickness and the trypanosomiases of man and animals from 1803 to March 31, 1909, and to recent papers on the tsetse-flies. The arrangement is by authors in alphabetical order, the papers being given again under the names of the journals in which they appeared. The price at which the book is published (4s. net) is only sufficient to cover part of the expense of printing.

Misses Ella and Florence Du Cane, who gave to the world last autumn a delightful book on "The Flowers and Gardens of Japan," have published, through Messrs. A. and C. Black, a similar volume on "The Flowers and Gardens of Madeira."

A new edition of "Manual of Military Ophthalmology," by M. T. Yarr, F.R.C.S.I., lieutenant-colonel Royal Army Medical Corps, member Ophthalmological Society of the United Kingdom, etc., is published by Messrs. Cassell and Company, Limited.

For reasons too obvious for comment the rivers and streams of England gather around their banks much of what is most beautiful of the various types of English scenery. And the happy idea has struck Messrs. Black to make them the subject of the next volume in their colour series. Mr. Sutton Palmer and Mr. A. G. Bradley collaborate in painting and describing most of the representative and characteristic rivers of the country, namely, those of the Welsh borderland, such as the Severn and Wye; those of Devon, such as the Dart, Tamar, Tavy, and Exe; while the rapid streams of Yorkshire, Derbyshire, and the Northern Border counties occupy other chapters. The slow-running rivers of the South and East of England are also dealt with.

